# Supplementary material for: Changes in anemia prevalence and the proportion of anemia associated with iron deficiency or inflammation in young children residing in Puno, Peru: Analysis using new World Health Organization guidelines for defining anemia
Source: PLoS One. 2026 Feb 20;21(2):e0342255. doi: 10.1371/journal.pone.0342255 (PMC12923139; doi:10.1371/journal.pone.0342255)
Supplement: Supplementary Material 1 — (DOCX) [file pone.0342255.s001.docx]

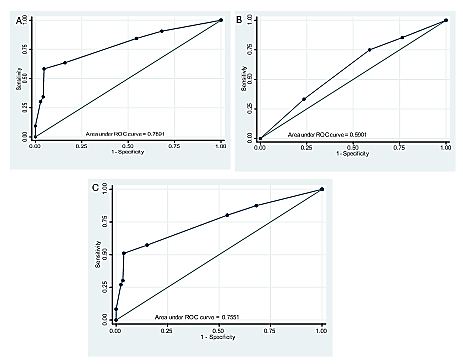
**Supplementary material 1.** Area under the curve adjusted for sex and age for the diagnosis of inflammatory anemia, using different IL-6 cutoff points: A. 50 pg/mL, B. 60 pg/mL, and C. 65 pg/mL.

**Supplementary Table 1.** Demographic and physiological parameters according to sex in young children aged 6-59 months from Puno, Peru

| **Variable** | **Females**  **(144)** | **Males**  **(166)** |
| --- | --- | --- |
| Age (months) | 34.5±0.9 | 34.56±1.0 |
| Altitude (meters) | 3769±37 | 3751±38 |
| Hb (g/dl) | 11.1±0.1 | 10.9±0.1 |
| Hepcidin (ng/ml) | 24.1±1.3 | 23.8±1.2 |
| EPO (mUI/ml) | 21.6±0.8 | 23.5±1.3 |
| sTfR (mg/L) | 0.7±0.03 | 0.8±0.04 |
| Serum Ferritin (ng/ml) | 18.9±0.9 | 22.1±2.2 |
| Interleukin-6 (pg/ml) | 45.6±1.7 | 46.4±1.7 |

Data are mean ± SD. P>0.05 between females and males in each of the variables studied. EPO: Erythropoietin. sTfR: Soluble transferrin receptor.

**Supplementary Table 2.** Iron status, EPO and Inflammatory markers in young children aged 6-

59 months in Puno, Peru.

| **Variable** | **Normal**  **Hb**  **(n= 179)** | **Mild**  **anemia**  **(n= 77)** | **Moderate**  **anemia**  **(n=50)** | **Severe**  **anemia**  **(n=4)** | **p-value** |
| --- | --- | --- | --- | --- | --- |
| Age (months) | 35.4 ± 0.9 | 33.7 ± 1.4 | 32.6 ± 1.7 | 35.3 ± 5.2 | 0.491 |
| Altitude (meters) | 3693 ± 41 | 3826 ± 39 | 3899 ± 27 | 3877 ± 27 | 0.060 |
| Hb (g/dL) | 11.9 ± 0.05 | 10.4 ± 0.04* | 9.09 ± 0.08* | 6.1 ± 0.30* | >0.001 |
| Hepcidin (ng/mL) | 23.4 ± 1.22 | 22.3 ± 1.4 | 29.6 ± 2.5 | 21.0 ± 3.3 | 0.055 |
| Epo (mIU/mL) | 22.1 ± 1.07 | 22.2 ± 1.1 | 25.1 ± 1.9 | 17.7 ± 2.3 | 0.440 |
| IL-6 (pg/mL) | 43.9 ± 1.51 | 48.2 ± 2.3 | 48.7 ± 3.7 | 53.4 ± 7.9 | 0.266 |
| sTfR (mg/L) | 0.7 ± 0.03 | 0.9 ± 0.06 | 0.9 ± 0.05 | 0.9 ± 0.2 | 0.090 |
| Ferritin (ng/ml) | 22.4 ± 1.93 | 18.3 ± 1.1 | 17.9 ± 1.6 | 14.4 ± 3.0 | 0.325 |

Diagnosis of normality and anemia based on current WHO criteria, WHO-2024. Data are mean ±SD. Data were analyzed by one-way ANOVA test. ANOVA Scheffe Test: Normal Hb vs mild, moderate and severe anemia *p<0.001.

**Supplementary Table 3.** Biomarkers in children aged 6-59 months form Puno classified as those with IL-6 >60 pg/mL and those with IL-6 ≤ 60 pg/mL

| **Variable** | **Group with**  **IL-6 > 60 pg/mL (n=67)** | **Group with**  **IL-6 ≤60 pg/mL (n=243)** | **p** |
| --- | --- | --- | --- |
| Age (months) | 33.4±1.8 | 33.43±0.9 | >0.05 |
| Altitude (meters) | 3588±99 | 3791±31 | >0.05 |
| Hb (g/dL) (WHO, 2001) | 10.9±0.2 | 10.8±0.1 | >0.05 |
| Hb (g/dL) (WHO, 2024) | 11.1±0.2 | 11.0±0.1 | >0.05 |
| Hepcidin (ng/mL) | 24.2±1.5 | 24.4±1.3 | >0.05 |
| Erythropoietin (mU/mL) | 25.4±3.4 | 21.5±0.7 | >0.05 |
| sTfR (mg/dL) | 0.8±0.1 | 0.7±0.03 | >0.05 |
| Serum Ferritin (ng/mL) | 22.3±2.0 | 20.7±1.8 | >0.05 |
| Interleukin-6 (IL-6) (pg/mL) | 76.7±2.2 | 35.7±0.9 | 0.001 |

Data are mean ±SD. Data were analyzed by T-student test.
