# Supplementary material for: Changes in anemia prevalence and the proportion of anemia associated with iron deficiency or inflammation in young children residing in Puno, Peru: Analysis using new World Health Organization guidelines for defining anemia
Source: PLoS One. 2026 Feb 20;21(2):e0342255. doi: 10.1371/journal.pone.0342255 (PMC12923139; doi:10.1371/journal.pone.0342255)
Supplement: Attachment — Submitted filename: Response to Reviewers.pdf [file pone.0342255.s002.pdf]

ANSWERS OF REVIEWERS

Manuscript ID: PONE-D-25-18153
Manuscript title: Changes in anemia and proportion of anemia associated with iron deficiency or inflammation in young children residing in Puno, Peru: Analysis using new World Health Organization guidelines for defining anemia

Dear Editor and Reviewers,

Thank you for the opportunity to revise and resubmit our article, "**Changes in anemia and proportion of anemia associated with iron deficiency or inflammation in young children residing in Puno, Peru: Analysis using new World Health Organization guidelines for defining anemia**" (PONE-D-25-18153). In our responses below, we have indicated where the corresponding changes were made in the revised manuscript. We are happy to address further questions that may arise.

We thank you very much for reviewing our revised submission. Please do not hesitate to contact us with any questions.

Journal Requirements

1. Please ensure that your manuscript meets PLOS ONE's style requirements, including those for file naming. The PLOS ONE style templates can be found at https://journals.plos.org/plosone/s/file?id=wjVg/PLOSOne_formatting_sample_main_body.pdf and https://journals.plos.org/plosone/s/file?id=ba62/PLOSOne_formatting_sample_title_authors_affiliations.pdf
Author response: We appreciate the Editor's input and guidance. We have ensured that our manuscript meets PLOS One's style requirements as outlined in the link included here.

2. Thank you for stating in your Funding Statement:
"The present study was funded by the Vice-Rectorate of Research of the Universidad Nacional del Altiplano, Puno, Peru. Dr. Rees was supported by the National Institutes of Health (K23HL173694)."

Please provide an amended statement that declares *all* the funding or sources of support (whether external or internal to your organization) received during this study, as detailed online in our guide for authors at http://journals.plos.org/plosone/s/submit-now. Please also include the statement "There was no additional external funding received for this study." in your updated Funding Statement.
Please include your amended Funding Statement within your cover letter. We will change the online submission form on your behalf.
Author response: We have added the following to the Funding statement as requested, "There was no additional external funding received for this study."

We have also added the amended Funding Statement in the cover letter.

3. Thank you for stating the following financial disclosure:
"The present study was funded by the Vice-Rectorate of Research of the Universidad Nacional del Altiplano, Puno, Peru. Dr. Rees was supported by the National Institutes of Health (K23HL173694)."

Please state what role the funders took in the study. If the funders had no role, please state: "The funders had no role in study design, data collection and analysis, decision to publish, or preparation of the manuscript."
If this statement is not correct you must amend it as needed.
Please include this amended Role of Funder statement in your cover letter; we will change the online submission form on your behalf.
Author response: We have added the following to the Funding Statement as requested, “The funders had no role in study design, data collection and analysis, decision to publish, or preparation of the manuscript.”

4. Thank you for stating the following in your Competing Interests section: “The authors declare no conflicts of interest.”

Please complete your Competing Interests on the online submission form to state any Competing Interests. If you have no competing interests, please state "The authors have declared that no competing interests exist.", as detailed online in our guide for authors at http://journals.plos.org/plosone/s/submit-now This information should be included in your cover letter; we will change the online submission form on your behalf.
Author response: We have updated the text as requested to now read, “The authors have declared that no competing interests exist.”

5. Thank you for uploading your study's underlying data set. Unfortunately, the repository you have noted in your Data Availability statement does not qualify as an acceptable data repository according to PLOS's standards.

At this time, please upload the minimal data set necessary to replicate your study's findings to a stable, public repository (such as figshare or Dryad) and provide us with the relevant URLs, DOIs, or accession numbers that may be used to access these data. For a list of recommended repositories and additional information on PLOS standards for data deposition, please see https://journals.plos.org/plosone/s/recommended-repositories.
Author response: We have uploaded the database to the ZENODO repository, which is listed among the Cross-disciplinary repositories permitted by PLOS One. In the manuscript, we have included both the DOI and the URL to ensure proper access and citation.

6. Your ethics statement should only appear in the Methods section of your manuscript. If your ethics statement is written in any section besides the Methods, please delete it from any other section.
Author response: We have added the following to the Methods section and removed the other mention of the Ethical Approvals: “The study was conducted in accordance with the Declaration of Helsinki and approved by the Institutional Review Board (or Ethics Committee) of Universidad Peruana Cayetano Heredia (protocol code N°464-20-19).”

7. If the reviewer comments include a recommendation to cite specific previously published works, please review and evaluate these publications to determine whether they are relevant and should be cited. There is no requirement to cite these works unless the editor has indicated otherwise.
Author response: Thank you for pointing this out. There were no such requests.

Reviewer #1:

Observation 1: The organization of the methods and results should be carefully reviewed and revised. I found this paper very confusing to read. Many sentences seemed out of place and belonged under other section headers, or seemed to repeat information that was already stated. For example, lines 195-197 seem like they belong in the subsequent section. Lines 204-213 seem like they should go under "prevalence of anemia by WHO Hb cutoffs". Lines 249-252 seem out of place in the section under ID and inflammation. There are several such instances.
Author response: We appreciate the Reviewer's comment. In response, we have carefully revisited the Results and have rearranged text to provide clearer writing and a more cohesive presentation of our findings as marked in tracked changes.

Observation 2: Clarify the terminology used to describe Hb and anemia before and after using the new WHO guidelines. Wording such as in lines 227-228 "lower in the group initially anemic who has normal Hb levels after being anemic" makes very little sense. These children are not changing anemia status in time, but are classified differently using different criteria. Please clarify throughout so that it's clear when you're referring to classification under the new guidelines versus old guidelines. Furthermore, be clear about whether you're referring to reclassification based on altitude or cut-off, as sometimes this wasn't clear.
Author response: We have carefully reviewed the entire manuscript and have updated the language from "changes" to "reclassification" and have clarified if the change in classification was done based on cut-offs or altitude adjustment as recommended by the Reviewer.

Observation 3: The discussion was repetitive. For example, lines 323-326, 342-348, and 366-370 say basically the same thing. It would be helpful to combine such statements into one concise paragraph.
Author response: We thank the Reviewer for this comment. We have consolidated these three sentences into a new paragraph in the Discussion.

Observation 4: There is almost no discussion of inflammation and its contribution to anemia in the discussion despite being a central focus of the results section. Please elaborate on how important inflammation is to anemia and potential causes of inflammation in children in this setting. Your abstract could be updated to reflect these changes.
Author response: We appreciate the Reviewer's comment. We have included the following in the Discussion in response to this comment, "The WHO attributes 50% of anemia cases to ID, 42% to inflammation, and 8% to other factors, including micronutrient deficiencies, hemoglobinopathies, and genetic disorders [28]. However, in Southern Peru—one of the regions with the highest anemia prevalence—these proportions appear different."

"A more comprehensive evaluation should include complete blood count parameters, such as mean corpuscular hemoglobin and volume, to assess ID, infection, or inflammation [38, 39]."

"Moreover, markers of iron status were measured, as well as inflammatory markers, which allowed the calculation of the fraction of anemia attributable to ID and to inflammation."

We have also included the following in the Abstract to emphasize our findings on inflammation: “The proportion of anemia due to ID was 27.5%, due to inflammation was 45.9%, …The new WHO guidelines did not substantially alter the estimated proportion of anemia associated with ID or inflammation.”

Observation 5: Lines 42: Should say “due to”
Author response: We have corrected this line in the Abstract as suggested. This line now reads, “The ratio of anemia due to iron deficiency (Ferritin <12 ng/mL) or inflammation (IL-6 >60 pg/mL) was estimated using adjusted Poisson regression models, reporting prevalence ratios (PR).”

Observation 6: Lines 89: Should say “of anemia among”
Author response: This has been corrected as requested. This line in the Introduction now reads, “Peru has one of the highest prevalences of childhood anemia throughout the Americas with historical estimates of anemia among young children aged 6 to 35 months as high as >70% in its highland regions and >40% across the country [7].”

Observation 7: Lines 106-107: Add a justification for your sample size. Was a sample size calculation done?
Author response: We have added the following to the Methods to describe our sample size. “Our sample size was determined by the convenience sample of 310 children that enrolled in our study. *Post hoc*, we determined that, at a significance level of 0.05 (two-sided) and a power of 80%, the minimum required sample size was 122 children per group (anemic and non-anemic) in order to detect a difference in the prevalence of anemia by the 2001 WHO guidelines and the 2024 WHO guidelines. The final recruited sample consisted of 310 children (179 non-anemic children and 131 anemic children).”

Observation 8: Lines 108-113: Please rewrite this sentence for clarity.
Author response: We have simplified this sentence in the Methods to read as follows, “We conducted our study in Puno, Peru since this region has one of the highest prevalences of anemia in the world (i.e., estimated 70% in children).”

Observation 9: Lines 128-133: Add section heading.
Author response: As suggested, we have added the following section heading “*Consent and Ethical Approvals*:”.

Observation 10: Lines 134-141: Suggest moving to the beginning of the methods.
Author response: We have moved the Study Setting section to follow the Study Design section as requested.

Observation 11: Lines 166-167: Justify the use of IL-6 > 60 pg/mL. The reference does not appear to mention this cut-off and does not seem to be appropriate for justifying this cut-off value.
Author response: Thank you for noting this. We have updated the reference to include a cutoff of IL-6 >60 pg/mL. We have also justified our use of a stricter cut-off for IL-6 in comparison to the referenced publication as follows in the Methods, “Prior studies have used an IL-6 value of >65 pg/mL to identify those with inflammation. However, as there is no reference standard for IL-6 levels in children, we applied a cutoff of >60 pg/mL in order to include more children with potential inflammation-mediated anemia.”

Observation 12: Lines 17-175: Did you adjust TBI for inflammation per BRINDA guidelines? See: 10.3945/ajcn.116.142307
Author response: Thank you for this comment and for including this reference. We used the same formula included in this publication. Additionally, thanks to the Reviewer, we have added this reference to the same section in the Methods.

Observation 13: Lines 227-228: This sentence is confusing. First, please reword per comment number 2 above. Second, based on your table, it looks like EPO was higher descriptively, but this was not statistically significant and is due to chance given your small n.
Author response: We thank the Reviewer for this comment. We have adjusted the wording in this sentence to now read, "Serum hepcidin, sTfR, EPO, and ferritin were similar among children with normal Hb, those with anemia, and those with normal Hb after classification with the 2024 WHO guidelines (Table 2)."

Observation 14: Line 231: Updated for altitude?
Author response: We thank the Reviewer for this comment. This line has been updated to read, "The prevalence of anemia with the 2011 WHO altitude adjustment correction was 50% in children aged 6-59 months, whereas with updated 2024 WHO recommended altitude adjustment correction the prevalence of anemia changed to 42%, resulting in a reduction of 8 percentage points (p=0.053)."

Observation 15: Line 236: Does Figure 1 provide updates based on both new cut-offs and altitude? Please clarify in text and figure legend.
Author response: We have added additional clarifying language in the legend for Figure 1 as suggested. This now reads, "Figure 1. Prevalence of anemia according to age groups 6 to 59, 6 to 23, and 24 to 59 months of age adjusted for 2001 WHO guidelines and 2024 WHO guidelines for both hemoglobin cutoff and altitude adjustment."

Observation 16: Lines 266-271: Please clarify how these proportions are different from the proportions given in lines 260-265.
Author response: We appreciate the opportunity to clarify these sentences in the Results. In response to the Reviewer, we have changed the text to read, "Among children aged 6-59 months without evidence of inflammation, the prevalence of IDA was 11.6% (16.1% among those aged 6-23 months and 10.0% for those aged 24-59 months [p>0.05]) (Table 3)." And "Among children aged 6-59 months, the prevalence of anemia due to inflammation without evidence of ID was 19.4% (17.3% among children aged 6-23 months and 20% among children aged 24-59 months; Table 3)."

Observation 17: Lines 272-274: Seems repetitive with earlier results (lines 249-252). What is the difference?
Author response: We have removed these lines as they were redundant with previous results.

Observation 18: Lines 342-345: Add the use of BRINDA adjustments to your methods. How did you adjust SF levels without the collection of CRP and AGP? Also, please justify why you did not use BRINDA for most of your estimates. This is the recommended methodology, but you seem to use it as a comparison. This should be made clearer.
Author response: The statistical technique applied was the same as that used in BRINDA (i.e., ≥1 marker of iron and ≥1 inflammatory marker). However, we could not fully apply the BRINDA approach as CRP and AGP were not collected in our study. We have added the following to the Limitations in response to the Reviewer's comment, "Although we used the approach outlined by BRINDA for TBI, we did not have data for CRP and AGP, so we were unable to adjust for inflammation according to this standard. However, we did use a proxy for adjustment of ≥1 marker of iron and ≥1 inflammatory marker."

Observation 19: Table 3: Define AF. Attributable fraction?
Author response: We have added a subheading in Table 3 as requested that defines AF as follows, "*Attributable Fraction (AF)".*

Observation 20: Table 3: I suggest separating the AF results from the others, either in another table or with headers, to clearly explain the difference between these prevalences.
Author response: We appreciate this recommendation and we have added a subheading to make this distinction clearer.

Observation 21: Table 4: Are these all run as separate models?
Author response: We appreciate the opportunity to provide additional clarification. This table includes unadjusted results. In response to the Reviewer's comment, we have changed the title of what is now Table 4 to read, "Unadjusted, bivariate analyses between the different candidate features and anemia as the outcome."

Reviewer #2:

Observation 1: Abstract-Lines 41-42: Improve writing to make it clearer that 6-23 and 24-59 months are brackets (subgroups) of the overall age interval (6-59 months). It is inferable, but it could be more objectively described.
Author response: We appreciate the Reviewer's comment. We have updated this text to read, "Applying the new WHO guidelines, anemia prevalence changed from 50% to 42.2% in children aged 6-59 months (62% to 47% in children aged 6-23 months and from 45.9% to 40.6% in children aged 24-59 months)."

Observation 2: Methods- Lines 120-126: The reference for CRED (15; Peruvian government site) indicates that the program is mandatory for all Peruvian children. If so, it would be better to report it. If not, does the children outside of the program not receive or are demanded a complete vaccination schedule? For non-Peruvians unfamiliar with the program, the description is a bit vague. It should either be a little bit clearer or just better summarized and referenced.
Author response: We thank the Reviewer for this observation. In response, we have changed the text to more accurately represent the nature of CRED visits as follows, "Routine CRED visits are mandatory and serve to protect children from diseases, detect any health risks early, as well as provide parents or caregivers with breastfeeding counseling, complementary feeding and other parenting issues [15]. CRED is a representative program in Peru, since it is mandatory and serves as the platform for routine vaccinations among children in Peru. During these visits, in addition to routine care, venous blood samples were drawn for Hb measurement and serum biomarkers for this study."

Observation 3: Lines 137-138: Beside the absolute number, consider adding the percentage of children in the Puno population, for perspective.
Author response: We thank the Reviewer for this comment. We have updated this text to read, "The Puno region has 13 provinces each one having several districts located at altitudes ranging from 610 to 4,660 meters above sea level [16] with a population of children aged 6-59 months of 40,162, which is approximately 1.7% of all children in this age group in Peru [17]."

Observation 4: Line 139: The word "maintained" gives the impression that they are financially supported, but it doesn't say by whom. Suggestion: "indigenous to", "inhabiting", or others.
Author response: In response to the Reviewer's comment, we have updated the text to

read, “The main ethnic group in Puno is a relatively homogeneous **ethnic** group called Aymara [18].”

Observation 5: Lines 157, 161, 173, 174 The sign before 4.5 g/dl, 3.3 g/dl and 4mg/kg (-) is a dash, a typo, minus/negative...?
Author response: We thank the Reviewer for providing us the opportunity to be more precise with our reporting. This has been changed to “ranging from subtracting 0 to 4.5 g/dl from the measured Hb level” on the first line.

Furthermore, for the mention of TBI, we have changed the text to read, “Total Body Iron (TBI) content was also calculated. TBI was <0 mg/Kg >-4 mg/Kg was ID and when TBI < -4 mg/Kg was IDA [23], using the formula [24]:”

Observation 6: Lines 154-164: An informative figure/table comparing the old and new criteria would be clearer and highly valuable for future consultation/citations.
Author response: We thank the Reviewer for this comment. We have created a new Table 1 to detail the requested information.

Observation 7: Line 170: The acronym IA is not previously presented in the manuscript. Inflammatory anemia?
Author response: We have added the following clarifying text to read, “inflammation-related anemia”.

Observation 8: Results
Lines 195-197: it says the non altitude-adjusted anemia prevalence was 5.2%. Line 204 says it was 50%. It is confusing.
Author response: We thank the Reviewer for pointing out the need to be very specific with our reporting. We have changed this language to read, “When Hb was not adjusted for altitude with any formula but by using WHO cutoffs, the prevalence of anemia in infants and children aged 6-59 months was 5.2%. This value was nearly eight-fold higher (42.2%) with Hb adjustment for altitude with the updated 2024 WHO guidelines and was 50% using the old WHO guidelines for altitude adjustment.”

Observation 9: Lines 209-214
A bar graph would be invaluable to show that one of the main changes with the new guideline, more than just the total prevalence, is the classification distribution, such as the increase in mild and decrease in moderate anemia.
Author response: We appreciate the Reviewer’s suggestion. We have created a new Figure 1 which demonstrates the prevalence of anemia using different categories of WHO guidelines. We have also deleted this information from Table 1 to avoid duplicating information.

Observation 10: Line 298
“Relationship” might be a false cognate in this context. Correlation.
Author response: We have changed the language as suggested. This line now reads, “No correlation was observed between serum IL-6 levels and serum hepcidin levels (R^2=0.072; r= 0.08; p>0.05).”

Observation 11: Discussion, Lines 303-305
“a greater reduction than achieved through years of universal iron supplementation”: as it is stated, it seems that updating the guidelines was a strategy to reduce the anemia prevalence and collaborated with it, when, in fact, it just changed the threshold. It was a reclassification.
Author response: We appreciate the Reviewer’s comment. In response, we have

updated this text to read, “Application of the updated WHO guidelines resulted in the reclassification of 15% of children aged 6-23 months as not having anemia who were previously classified as having anemia by previous WHO guidelines.”

Observation 12: Table 1
“Quantitative variables expressed (age, hemoglobin and biomarkers) as mean ± SD”. Rewrite as “Quantitative variables (age, hemoglobin and biomarkers) expressed as mean ± SD”
Author response: We have changed this footnote as requested.

Observation13: Table 2
The line N should be inside the header line, such as: Normal Hb (n=155); Anemic (n=131); Normal Hb after reclassification (n=24).
“normal after update cutpoint children”: grammar is confusing. Maybe “normal after reclassification”?
Author response: We thank the Reviewer for these suggestions. We have updated the Table as recommended.
