# Supplementary material for: Changes in anemia prevalence and the proportion of anemia associated with iron deficiency or inflammation in young children residing in Puno, Peru: Analysis using new World Health Organization guidelines for defining anemia
Source: PLoS One. 2026 Feb 20;21(2):e0342255. doi: 10.1371/journal.pone.0342255 (PMC12923139; doi:10.1371/journal.pone.0342255)
Supplement: Attachment — Submitted filename: ANSWER TO REVIEWER PLOS ONE 27.11.docx [file pone.0342255.s003.docx]

**Manuscript ID:** PONE-D-25-18153R1

**Tittle:** Changes in anemia prevalence and the proportion of anemia associated with iron deficiency or inflammation in young children residing in Puno, Peru: Analysis using new World Health Organization guidelines for defining anemia

Dear Editor and Reviewers,

Thank you for the opportunity to revise and resubmit our article. In our responses below, we have indicated where the corresponding changes were made in the revised manuscript. We are happy to address further questions that may arise.

**Reviewer #1: The authors addressed several of my prior comments; however, the organization within the text, as well as the definitions of iron-deficiency anemia, inflammation-mediated anemia, and other categorizations, are not well-defined and difficult to follow in the text. Additionally, the use of IL-6 for inflammation-mediated inflammation, which lacks a validated cut-off, is problematic.**

**ANSWER:** We appreciate the Reviewer’s comments and the time they spent reviewing our manuscript again. We have addressed each of these comments in detail as outline below. We believe that the incorporation of their input has greatly strengthened the reporting of our work.

**Observation 1. While I appreciate the authors' efforts to improve organization, significant issues with clarity and structure remain. The results are misplaced within the subheadings, and there is still repetition.
a. Lines 184-186 and 204-208 report nearly identical unadjusted anemia prevalence (5.2% vs 5.1%). Are these referring to the same value? Please remove this unnecessary redundancy if so.**

**ANSWER:** These values were the same. In response to the Reviewer’s comment, we have removed the redundancy.

**b. The section "Prevalence of Anemia by Altitude Hb Correction Factor" (lines 203-214) largely repeats information from the preceding section and should be consolidated or deleted.**

**ANSWER:** The structure of the paragraphs has been modified and redundant content has been removed (i.e., this section was removed as recommended by the Reviewer).

**c. Lines 249-253 (BRINDA method results) appear disconnected from the surrounding content and need better integration or explanation**
**ANSWER:** All sections referring to the BRINDA method have been removed in response to other comments below.

**Observation 2. Justification of the methods used for IL-6 and the cut-off chosen in lines 155-158. Your explanation to artificially adjust the cut-off to “include more children with potential inflammation-mediated anemia” is not scientifically justifiable.
a. It is not appropriate to arbitrarily create a cut-off value and falsely inflate the number of children with inflammation.**

**ANSWER**: The criteria for using the selected cutoff point have been detailed in the manuscript in the Methods on page 7 as described here: “*We used IL-6 to measure inflammation [21, 22]. As prior studies have used varying cutoffs of IL-6, our selected cutoff was based on the 75th percentile, which corresponds to the cutoff of IL-6>60 pg/mL. We assessed the optimal IL-6 level for the diagnosis of inflammation-mediated anemia, which demonstrated an area under the receiver operating characteristic curve of 0.79; 95% CI: 0.71-0.81 (Supplementary Material 1). Thus, an IL-6 level of >60 pg/mL was used for our analyses.”*

| IL-6 | Inflammation anemia | |
| --- | --- | --- |
|  | AUC* | 95%CI |
| 50 pg/mL | 0.59 | 0.50-0.68 |
| 60 pg/mL | 0.76 | 0.71-0.81 |
| 65 pg/mL | 0.73 | 0.68-0.79 |
| *Area under the curve adjusted for sex and age. | | |

***Supplementary material 1.*** Area under the curve adjusted for sex and age for the diagnosis of inflammatory anemia, using different IL-6 cutoff points: A. 50 pg/mL, B. 60 pg/mL, and C. 65 pg/mL.

**b. You cite two studies that use >65 pg/ml, but one of these studies doesn’t use this cut-off and the other appears to use a cut-off of 70 pg/ml. Please clarify. I found one paper that used a cut-off of 50 pg/ml:** [**https://bmcnutr.biomedcentral.com/articles/10.1186/s40795-023-00748-3#Sec2**](https://bmcnutr.biomedcentral.com/articles/10.1186/s40795-023-00748-3#Sec2)**.**

**ANSWER:** We appreciate the heterogeneity in reporting of IL-6 cutoffs in prior studies. In response to the Reviewer’s input, we have performed additional analyses as above to determine the diagnostic capacity of IL-6 based on different cut-off points. In the previous response, we detailed the areas under the curve for the 50, 60, and 65 pg/mL points. We have also included this as part of the supplementary material.

**c. An option is to use one of these cut-offs from the literature as the main analysis, based on which is most appropriate to your study population, and then conduct a sensitivity analysis at a different threshold and report how these results change your interpretation of the proportion of inflammation and inflammation-mediated anemia. Detailed in the previous response.**

**ANSWER:** We have considered the calculation of inflammation prevalence using the cut-off points of 50 and 60 pg/mL reported in Table 2 (36.8% vs. 21.6%, respectively). We have also performed a comparative AUC analysis to determine which of the cut-off points is optimal for the diagnosis of inflammation-mediated anemia.

**d. It’s essential to note the use of IL-6, rather than CRP and AGP, as a limitation of your study, since there is no established cut-off for IL-6 to determine inflammation-mediated anemia. This limits the comparability of your work to other studies.**

**ANSWER**: Certainly, we have included in the discussion section, the use of Il-6 rather than CRP and AGP as a limitation that limit comparisons with other studies using these inflammatory markers.

*“*Furthermore, we cannot perform comparative analyses with studies that considered inflammatory biomarkers such as those applied by the BRINDA method (e.g., CRP and AGP), as these were not measured…”

**Observation 3. In the methods, please clearly write how you define iron-deficiency anemia, inflammation-mediated anemia, and the use of the BRINDA approach. (lines 158-162 and 170-172)
a. I assume in your inflammation-mediated anemia definition, you meant to include an IL-6 cut-off. Add this. Also include whether this is regardless of ferritin level.**

**ANSWER:** The following has been incorporated into the text: “*Iron deficiency anemia (IDA) was defined as Hb <10.5 g/dL and ferritin below 12 ng/mL for those aged 6-23 months and <11 g/dL and ferritin below 12 ng/mL for those aged 24-59 months. For IDA without inflammation, the criterion of IDA and IL-6≤60 pg/mL was used. Inflammation without IDA was defined as IL-6 >60 pg/mL with normal Hb. Inflammation with IDA was defined as IL-6 >60 pg/mL with IDA.”*

**b. Keep consistent terminology. If you use** **inflammation-mediated anemia, then don’t change to inflammation-related anemia, as this just created confusion.**
**ANSWER:** We appreciate the opportunity to provide added clarity. We have reviewed the manuscript and we now exclusively use “inflammation-mediated anemia”

**c. How did you use BRINDA if you didn’t measure CRP and AGP? Is this justifiable?**

**ANSWER:** Since BRINDA uses CRP and AGP, the BRINDA method analysis in our manuscript has been removed. Table 1 shows the hemoglobin cut-off points, and Table 2 and the methodology show the cut-off points for the iron and inflammation biomarkers used.

**Observation 4.** **Inconsistency between prevalence of ID.**
a. Lines 216 state ID prevalence is 24.2%, but lines 231-232 state it's 3.5%. Clarify why these are different.

**ANSWER:** Line 216 refers to general iron deficiency assessed in the entire sample (i.e., in the 311 children who had ferritin below the cutoff point [<12 ng/mL]). Within this population, there were both anemic (Hb below threshold) and non-anemic children. In order to minimize potential for confusion, lines 231-232 have been removed because the key results are those related to IDA.

**Observation 5.** **Inconsistency in results using your cut-off of inflammation-mediated anemia versus the BRINDA method.**
**a. In lines 241-242, the “proportion of anemia associated with inflammation was 45.9%” and in lines 249-250 “the proportion of anemia from ID and inflammation according to the BRINDA method was 11% and 2.1%”. There is also a huge inconsistency with the proportion attributable to ID between your results and using the BRINDA approach (27.% vs 11%).**

**ANSWER:** The analysis of the BRINDA method has been removed as above.

**b. Explain the 2.5-fold difference in ID attribution (11% vs 27.5%) and the 22-fold difference in inflammation attribution (2.1% vs 45.9%) between methods.**

**ANSWER:** These were the values obtained using the BRINDA method, which have been removed from the manuscript, leaving only the values calculated based on our measured markers. The attributable fractions would be 27.5% and IA 45.9%.

**c. Decide which of these will be the primary method and why. In the discussion, you state, “In our study, only 11% of anemia cases were attributable to ID, while 87% were linked to other causes.” (lines 298-299). This makes it seem like you are using the most dramatic of these two results to frame your discussion.**

**ANSWER:** In response to the Reviewer, it has been specified that the prevalence of ID was 11% in the total population as follows: *“**In our study, only 11% of anemia cases were due to ID overall. However, ID as an attributable factor in 45.9% of the anemic population...”*

**d. You then state in lines 307-309 that 27.5% of anemia is associated with ID, directly contradicting the paragraph before. Clarify.**

**ANSWER:** The value of 27.5% corresponds to the attributable factor, as specified in the text, which differs from the prevalence since the prevalence considers the entire child population evaluated and the attributable factor only considers children diagnosed with anemia.

**Observation 6.** **Lines 270-271, unclear why you included an equation here. If describing correlation, using R2.**

**Answer:** Thanks for the comment. We have removed the equation and only R2 is now displayed.

**Observation 7.** **The sentence in lines 292-294 is confusing. As written, it sounds as if these proportions are from an original report from the WHO and then the updated WHO guidelines, but the updated guidelines did not do attribution of causes. If this 26.2% comes from your results, then be clear about that and do not add a citation to this number. Also, if you are specifying all causes beside ID, it should be 26.6%+45.9% based on your results.**

**Answer:** Thank you for your comment. What we wanted to point out is that although we report attributable fractions without considering ID, the group of anemic patients used in the calculation differs due to the update of the Hb cutoff point. We have changed the following part of the paragraph for better understanding:

*“50.2% of anemia cases were attributed to non-ID causes using the old WHO Hb cutoff. [6] Conversely, 26.6% of anemia cases were attributed to non-ID causes when the current Hb cutoff was used. [2]”*

**Observation 8.** **In the strengths, you write that you measured inflammatory markers, as plural. But as I understand it you only measured one inflammatory marker, IL-6.**

**Answer:** Thank you for pointing that out, it has been changed.

**Observation 9.** **Lines 350-351, the used of the updated WHO guidelines is noted as a strength. This does not seem like a strength to me if the aim of the paper is to compare the old versus new guidelines.**
**Answer:** Thank you for pointing that out. This text has been removed as a result of this recommendation.

**Observation 10.** **Lines 357-358. I don’t think this is a valid rebuttal to this limitation. You did not use CRP and AGP, limiting the use of the BRINDA method, and the inflammatory marker you did use does not have a validated threshold. This must be included in the limitations.**

**Answer:** All mention of the BRINDA method has been removed from our manuscript in response to the Reviewer’s comments.

**Observation 11.** **Table 4 requires clarification:
a. The row "Anemia associated with ID" (31.3%) is undefined and doesn't correspond to any other reported value. Please clarify what this represents.**

**ANSWER:** The row on anemia associated with ID has been removed, as this is included in the total anemia prevalence.

**b. The attributable fractions don't sum to 100%. The text mentions 26.6% "other causes" but this isn't shown in the table.**

**ANSWER:**  The specific percentage of the other factors has been calculated and included in Table 4, with a total sum of 100% for each age group. It has also been specified that genetic alterations, deficiencies of other vitamins, and alterations due to environmental pollution were not specifically measured. They were only mentioned in general terms in the text.

**c. The opposite age-pattern results between methods (BRINDA: younger children have more inflammation; your IL-6 method: older children have more inflammation) needs explanation.**

**ANSWER:** The section of the text that mentioned the BRINDA method has been removed.

**Reviewer #2:** **The majority of recommendation were addressed by the author. A few still lingers, as described below.**

**Observation 12. Table 2 “normal after update cutpoint children” was not corrected in the footnote. Change it to “normal after reclassification”**

**Answer:** Thank you for pointing that out. This has been changed as recommended.

**Observation 13.** **Figure 1 Although I do understand that it is a matter of choice from the authors, in my perception, severe/moderate/mild reclassification changes are better visualized in a piled bars chart. It will not include the changes in total prevalence though. Also, the percentage values (59%, 46,4%…) should be plotted (this recommendation applies for other charts too).**

**Answer:**

Figure 1. Prevalences of anemia in infants aged 6 to 59 months residing in Puno, by category. Black bar, mild anemia. Black lines bar, moderate anemia. And, gray bar, severe anemia calculated based on old and new WHO guidelines.

We have kept Figure 2 because the arrangement of the bars allows us to make statistical comparisons more easily. Figure 3 was removed because it contains information described in Table 4.

**Observation 14.** **Figure 2 The legend “Anemia 2001/Anemia 2024” gives the impression that there were a temporal analysis, in different years, when it was just the year of the guidelines. Correct it for clarity.**

**Answer:** Thank you for pointing that out. In response, it has been changed accordingly.
