# Supplementary material for: Changes in anemia prevalence and the proportion of anemia associated with iron deficiency or inflammation in young children residing in Puno, Peru: Analysis using new World Health Organization guidelines for defining anemia
Source: PLoS One. 2026 Feb 20;21(2):e0342255. doi: 10.1371/journal.pone.0342255 (PMC12923139; doi:10.1371/journal.pone.0342255)
Supplement: Attachment — Submitted filename: Answer to reviewers 10.12.docx [file pone.0342255.s004.docx]

**Manuscript ID:** PONE-D-25-18153R1

**Tittle:** Changes in anemia prevalence and the proportion of anemia associated with iron deficiency or inflammation in young children residing in Puno, Peru: Analysis using new World Health Organization guidelines for defining anemia

Dear Editor and Reviewers,

Thank you for the opportunity to revise and resubmit our article. In our responses below, we have indicated where the corresponding changes were made in the revised manuscript. We are happy to address further questions that may arise.

**Reviewer #1: The authors addressed several of my prior comments; however, the organization within the text, as well as the definitions of iron-deficiency anemia, inflammation-mediated anemia, and other categorizations, are not well-defined and difficult to follow in the text. Additionally, the use of IL-6 for inflammation-mediated inflammation, which lacks a validated cut-off, is problematic.**

**ANSWER:** We appreciate the Reviewer’s comments and the time they spent reviewing our manuscript again.

We thank the reviewer for the valuable and constructive comments. In response, we have revised the manuscript to improve the organization and clarity of the anemia classifications and to explicitly define each category, as requested.

**1. Clarification and definitions of anemia categories**
We have added clear operational definitions in the Methods section to ensure transparency and facilitate interpretation. The revised definitions are now as follows:

- Iron-deficiency anemia (IDA):
  - For children aged 6–23 months: Hb < 10.5 g/dL and ferritin < 12 ng/mL
  - For children aged 24–59 months: Hb < 11 g/dL and ferritin < 12 ng/mL
- IDA without inflammation:
  - Meets criteria for IDA and IL-6 ≤ 60 pg/mL
- Inflammation without IDA:
  - IL-6 > 60 pg/mL with normal Hb levels
- Inflammation with IDA:
  - IL-6 > 60 pg/mL and meets criteria for IDA

These definitions have now been integrated into a reorganized subsection to improve readability and logical flow.

**2. Use of IL-6 as an indicator of inflammation**
We acknowledge the reviewer’s concern regarding the lack of a universally validated IL-6 cut-off for defining inflammation. In the revised manuscript, we now explicitly state this limitation. IL-6 was used as a *supportive biomarker* based on its well-established role in inflammatory pathways and hepcidin regulation. We have clarified that the threshold of 60 pg/mL was used for analytical categorization in this study but is not a standardized diagnostic cut-off. This clarification has been added to both the Methods and Discussion sections.

We trust that these revisions adequately address the reviewer’s concerns and improve the clarity and rigor of the manuscript.

**Observation 1. While I appreciate the authors' efforts to improve organization, significant issues with clarity and structure remain. The results are misplaced within the subheadings, and there is still repetition.
a. Lines 184-186 and 204-208 report nearly identical unadjusted anemia prevalence (5.2% vs 5.1%). Are these referring to the same value? Please remove this unnecessary redundancy if so.**

**ANSWER:** The correct value is 5.2%. A variation in the value was generated due to the rounding of decimals. Likewise, the structure has been improved and now it is only reported in one section (lines 185-186): “When Hb was not adjusted for altitude with any formula, the prevalence of anemia in infants and children aged 6-59 months was 5.2%...”

**b. The section "Prevalence of Anemia by Altitude Hb Correction Factor" (lines 203-214) largely repeats information from the preceding section and should be consolidated or deleted.**

**ANSWER:** The structure of the section has been revised, and redundant content has been removed. As recommended by the reviewer, the subsection “Prevalence of Anemia by Altitude Hb Correction Factor” has been deleted. Its essential information has been integrated and summarized within the paragraph on Prevalence of Anemia using WHO Hb cutoffs.

**c. Lines 249-253 (BRINDA method results) appear disconnected from the surrounding content and need better integration or explanation**
**ANSWER**: All sections referring to the BRINDA method have been removed in response to this and other related comments. Because C-reactive protein (CRP) and α-1-acid glycoprotein (AGP) were not measured in our study, the BRINDA adjustment could not be appropriately applied. Instead, we used interleukin-6 (IL-6) as the inflammatory marker, given its direct involvement in hepcidin induction. This approach is physiologically relevant because hepcidin-mediated iron sequestration is a central mechanism of inflammation-induced anemia, and IL-6 is a key upstream regulator of this pathway.

**Observation 2. Justification of the methods used for IL-6 and the cut-off chosen in lines 155-158. Your explanation to artificially adjust the cut-off to “include more children with potential inflammation-mediated anemia” is not scientifically justifiable.
a. It is not appropriate to arbitrarily create a cut-off value and falsely inflate the number of children with inflammation.**

**ANSWER**: We appreciate the reviewer’s important comment regarding the justification for the IL-6 cut-off. In response, we have substantially revised the Methods section and expanded the explanation to ensure that our approach is scientifically rigorous and clearly presented.

First, IL-6 was selected because prior studies have used multiple IL-6 thresholds to define inflammation, and there is no universally accepted or validated cut-off for inflammation-mediated anemia in young children. For this reason, we evaluated several IL-6 levels within our dataset to determine which threshold best identified inflammation-associated anemia.

Second, rather than arbitrarily choosing a cutoff, we performed a receiver operating characteristic (ROC) analysis to empirically determine the IL-6 level with the best diagnostic performance. This analysis showed that IL-6 > 60 pg/mL had the optimal discriminatory capacity:

| **IL-6 cutoff** | **AUC*** | **95% CI** |
| --- | --- | --- |
| 50 pg/mL | 0.59 | 0.50–0.68 |
| 60 pg/mL | 0.76 | 0.71–0.81 |
| 65 pg/mL | 0.73 | 0.68–0.79 |

*Adjusted for sex and age.

These results (now presented in Supplementary Material 1) show that 60 pg/mL provided the highest AUC and therefore the best balance of sensitivity and specificity. Based on this empirical evidence, IL-6 > 60 pg/mL was selected for the final analysis.

Third, IL-6 is directly implicated in the pathophysiology of inflammation-mediated anemia because it upregulates hepcidin, which restricts iron availability for erythropoiesis. This mechanistic relevance provides an additional biological rationale for using IL-6 in the classification.

We hope that this clarification demonstrates that our IL-6 threshold was not arbitrarily defined, but rather derived from data-driven analysis, supported by biological plausibility, and grounded in methodological rigor.

***Supplementary material 1.*** Area under the curve adjusted for sex and age for the diagnosis of inflammatory anemia, using different IL-6 cutoff points: A. 50 pg/mL, B. 60 pg/mL, and C. 65 pg/mL.

**b. You cite two studies that use >65 pg/ml, but one of these studies doesn’t use this cut-off and the other appears to use a cut-off of 70 pg/ml. Please clarify. I found one paper that used a cut-off of 50 pg/ml:** [**https://bmcnutr.biomedcentral.com/articles/10.1186/s40795-023-00748-3#Sec2**](https://bmcnutr.biomedcentral.com/articles/10.1186/s40795-023-00748-3#Sec2)**.**

**ANSWER:** We thank the reviewer for pointing out the discrepancy regarding IL-6 cutoffs in the cited literature. We have now carefully re-examined the studies and clarified their thresholds more accurately.

1. Choque-Quispe et al. (2024) used IL-6 > 70 pg/mL as the threshold for defining inflammation in children.
2. Rincón-López et al. (2021) did not use a cutoff of 65 pg/mL. Instead, IL-6 among ten proinflammatory markers was evaluated for diagnostic performance in children with sickle-cell disease, where the study identified IL-6 ≈ 125 pg/mL as an optimal discriminator for bacterial infection. This value is not intended as a general inflammation cutoff for anemia-related analyses.

The reviewer also provided a relevant study using IL-6 > 50 pg/mL (BMC Nutrition 2023), which we now cite appropriately. This confirms that the literature contains multiple non-standardized IL-6 thresholds (50, 60, 70, 125 pg/mL), depending on the population, clinical context, and analytical purpose.

To avoid any inconsistency and ensure methodological rigor, we adopted a data-driven approach:

- We conducted a ROC analysis within our dataset to determine the threshold with the best diagnostic performance for inflammation-mediated anemia.
- IL-6 > 60 pg/mL showed the highest AUC (0.76; 95% CI: 0.71–0.81), outperforming 50 pg/mL and 65 pg/mL.
- Therefore, IL-6 > 60 pg/mL was selected for analyses specifically related to inflammation-mediated anemia.
- For the calculation of the overall prevalence of inflammation, we additionally included the literature-based threshold IL-6 > 50 pg/mL, as cited by the reviewer.

These clarifications have been incorporated into the revised Methods section, and all IL-6–related citations have been corrected accordingly.

We hope that this detailed clarification resolves the reviewer’s concerns and strengthens the methodological transparency of the manuscript.

Accordingly, the updated Methods section now reads:

“*We used IL-6 to assess inflammation [21,22]. Because previous studies have applied different IL-6 thresholds (including >50 pg/mL, >70 pg/mL, and higher values depending on clinical context), and no standardized cutoff exists for defining inflammation-mediated anemia in young children, we evaluated several IL-6 levels within our dataset. Receiver operating characteristic (ROC) analysis identified 60 pg/mL as the optimal cutoff for detecting inflammation-mediated anemia (AUC: 0.76; 95% CI: 0.71–0.81; Supplementary Material 1), outperforming 50 pg/mL and 65 pg/mL. Therefore, IL-6 > 60 pg/mL was used to classify inflammation-mediated anemia and to estimate the fraction of anemia attributable to inflammation. In addition, for determining the overall prevalence of inflammation, we also applied the literature-based threshold of IL-6 > 50 pg/mL [23*]”.

**c. An option is to use one of these cut-offs from the literature as the main analysis, based on which is most appropriate to your study population, and then conduct a sensitivity analysis at a different threshold and report how these results change your interpretation of the proportion of inflammation and inflammation-mediated anemia. Detailed in the previous response.
ANSWER:** We agree with the reviewer and have implemented this recommendation. Specifically, we evaluated inflammation prevalence using the two IL-6 thresholds most relevant to our study population: 50 pg/mL (literature-based) and 60 pg/mL (data-driven). As shown in Table 2, the estimated prevalence of inflammation differed between these cutoffs (36.8% vs. 21.6%, respectively).

To determine which threshold was most appropriate for identifying inflammation-mediated anemia, we additionally conducted a comparative ROC analysis (Supplementary Material 1). IL-6 > 60 pg/mL demonstrated the highest diagnostic performance (AUC: 0.76; 95% CI: 0.71–0.81), outperforming both 50 pg/mL and 65 pg/mL.

Based on these results:

- IL-6 > 60 pg/mL was used in the *main analysis* for identifying inflammation-mediated anemia and estimating the attributable fraction.
- IL-6 > 50 pg/mL was included as a *sensitivity analysis* for estimating the overall prevalence of inflammation.

This dual approach increases transparency and ensures that the interpretation of our findings is robust to different IL-6 thresholds.

**d. It’s essential to note the use of IL-6, rather than CRP and AGP, as a limitation of your study, since there is no established cut-off for IL-6 to determine inflammation-mediated anemia. This limits the comparability of your work to other studies.**

**ANSWER**: We appreciate this important observation, and we have expanded our discussion of this limitation. Because CRP and AGP were not measured, we could not apply BRINDA-based inflammation adjustments. Instead, IL-6 was used as an inflammatory marker due to its direct mechanistic role in hepcidin induction. However, we explicitly acknowledge that no universally established IL-6 cutoff exists for defining inflammation-mediated anemia, which limits direct comparability with studies using CRP and AGP.

The Discussion section now includes the following text:

*“A limitation of our study is the use of IL-6, rather than CRP and AGP, to assess inflammation, as no standardized IL-6 threshold exists for defining inflammation-mediated anemia. Consequently, our results cannot be directly compared with studies applying BRINDA adjustments or based on CRP/AGP-defined inflammation.
However, the use of IL-6 also represents a strength of our analysis, as IL-6 is a key upstream regulator of hepcidin, the central hormone responsible for iron sequestration during inflammation. Because hepcidin-mediated iron restriction is the primary mechanism underlying inflammation-induced anemia, IL-6 provides a biologically relevant and mechanistically aligned indicator of inflammatory activity.
Moreover, IL-6 responds rapidly to inflammatory stimuli and is less influenced by chronic, low-grade processes compared to CRP or AGP, allowing more precise identification of acute inflammatory states in young children”.*

This clarification strengthens methodological transparency and aligns the manuscript with the reviewer’s recommendation.

**Observation 3. In the methods, please clearly write how you define iron-deficiency anemia, inflammation-mediated anemia, and the use of the BRINDA approach. (lines 158-162 and 170-172)
a. I assume in your inflammation-mediated anemia definition, you meant to include an IL-6 cut-off. Add this. Also include whether this is regardless of ferritin level.**

**ANSWER:** We thank the reviewer for this important clarification request. The Methods section has been revised to explicitly define iron-deficiency anemia (IDA), inflammation-mediated anemia (including the IL-6 cutoff), and the handling of the BRINDA approach.

Specifically:

1. Iron-deficiency anemia (IDA) was defined using age-specific hemoglobin and ferritin thresholds, consistent with WHO recommendations.
2. Inflammation-mediated anemia is now explicitly defined as the coexistence of anemia and inflammation, the latter measured using IL-6. An IL-6 level >60 pg/mL was used based on ROC analysis, and this definition applies regardless of ferritin level, because ferritin is elevated during inflammation and cannot validly classify IDA in the presence of inflammatory activity.
3. Regarding BRINDA, we have clarified in the Methods that the BRINDA approach could not be applied, as CRP and AGP were not measured. For transparency, this is now stated directly.

These definitions are now clearly presented in the revised Methods section, reproduced below for clarity.

**b. Keep consistent terminology. If you use** **inflammation-mediated anemia, then don’t change to inflammation-related anemia, as this just created confusion.**
**ANSWER:** We appreciate the reviewer’s comment regarding terminology consistency. We have thoroughly reviewed the manuscript, and all instances of the term *“inflammation-related anemia”* have been revised to ensure exclusive use of the term “*inflammation-mediated anemia*” throughout. This standardization avoids ambiguity and maintains terminological coherence across all sections of the manuscript.

**c. How did you use BRINDA if you didn’t measure CRP and AGP? Is this justifiable?**

**ANSWER:** We thank the reviewer for this important clarification. The BRINDA approach requires measurements of C-reactive protein (CRP) and α1-acid glycoprotein (AGP) to adjust iron biomarkers for inflammation. Because our study did not include CRP or AGP measurements, the BRINDA method cannot be applied. For this reason, all BRINDA-related analyses and references have been entirely removed from the manuscript.

To ensure transparency, Table 1 now presents the hemoglobin cutoffs used, and Table 2 together with the Methods section clearly describe the iron and inflammation biomarkers employed in our analysis (ferritin and IL-6) and their corresponding thresholds. We have also explicitly stated that the absence of CRP and AGP limits comparability with studies using BRINDA adjustments.

**Observation 4.** **Inconsistency between prevalence of ID.**
a. Lines 216 state ID prevalence is 24.2%, but lines 231-232 state it's 3.5%. Clarify why these are different.

**ANSWER:** We appreciate the reviewer’s observation and thank you for the opportunity to clarify this point. The two values refer to different denominators, which is why they are not directly comparable.

- The value reported in line 216 (24.2%) refers to overall iron deficiency (ID) in the full sample, defined as ferritin <12 ng/mL.
- The value in lines 231–232 (3.5%) referred specifically to iron deficiency (ID) among children classified with inflammation-mediated anemia, not the full sample.

Because this second percentage represents a subgroup-specific estimate, its inclusion created confusion and did not contribute meaningfully to the main results. Therefore, we have removed the 3.5% value from the Results section to maintain clarity. The manuscript now consistently reports the overall prevalence of iron deficiency (ID) and iron-deficiency anemia (IDA), which are the key outcomes.

**Observation 5.** **Inconsistency in results using your cut-off of inflammation-mediated anemia versus the BRINDA method.**
**a. In lines 241-242, the “proportion of anemia associated with inflammation was 45.9%” and in lines 249-250 “the proportion of anemia from ID and inflammation according to the BRINDA method was 11% and 2.1%”. There is also a huge inconsistency with the proportion attributable to ID between your results and using the BRINDA approach (27.% vs 11%).**

ANSWER: We thank the reviewer for pointing out this inconsistency. As noted in earlier responses, all analyses based on the BRINDA method have now been completely removed from the manuscript because CRP and AGP levels were not measured and BRINDA cannot be validly applied. With the BRINDA-based calculations removed, the inconsistencies between the two sets of results no longer exist.

The revised Results section now reports only the findings derived from our validated definitions of iron-deficiency anemia (IDA) and inflammation-mediated anemia. Specifically, we present the prevalence of inflammation and the proportion of anemia attributable to inflammation based solely on IL-6–defined inflammation. The key result is:

“*The attributable fraction of inflammation-mediated anemia was 45.9%, while 26.6% of anemia was attributable to other non–iron-related causes (e.g., genetic, environmental factors) (Table 4)*.”

By removing the BRINDA-derived values (11% and 2.1%), the manuscript now contains a single, internally consistent set of estimates, avoiding confusion and ensuring methodological coherence.

**b. Explain the 2.5-fold difference in ID attribution (11% vs 27.5%) and the 22-fold difference in inflammation attribution (2.1% vs 45.9%) between methods.**

**ANSWER:** The large differences in the proportions attributed to iron deficiency and inflammation were derived from the BRINDA-adjusted estimates. As the BRINDA method requires CRP and AGP values, which were not measured in our study, these results were not methodologically justifiable. Therefore, all BRINDA-derived calculations have been removed from the manuscript.

Only the results based on our directly measured biomarkers (ferritin and IL-6) are retained. Using these validated markers, the attributable fractions are:

- 27.5% for iron-deficiency anemia, and
- 45.9% for inflammation-mediated anemia.

These values now reflect a consistent analytic approach aligned with the biomarkers actually measured in our population.

**c. Decide which of these will be the primary method and why. In the discussion, you state, “In our study, only 11% of anemia cases were attributable to ID, while 87% were linked to other causes.” (lines 298-299). This makes it seem like you are using the most dramatic of these two results to frame your discussion.**

**ANSWER:** We appreciate this important observation. To ensure clarity and methodological consistency, we now explicitly distinguish between:

1. Prevalence of iron deficiency (ID) in the total population:
   This refers to ferritin <12 ng/mL *regardless of anemia status*, which in our study was 11%.
2. Attributable fraction among children with anemia:
   This reflects the proportion of anemia cases attributable to a given factor based on our measured biomarkers.
   - Iron deficiency–mediated anemia: 27.5%
   - Inflammation-mediated anemia (based on IL-6 >60 pg/mL): 45.9%

To avoid confusion between population prevalence and attributable anemia fractions, the Discussion has been rewritten to clarify that:

“*Iron deficiency was present in 11% of the total population. However, among children with anemia, iron deficiency accounted for 27.5% of cases, while inflammation-mediated anemia accounted for 45.9%*.”

We further clarify that the BRINDA-derived estimates were removed, as CRP and AGP, the biomarkers required for BRINDA corrections were not measured. Therefore, the primary method used in this study is based solely on ferritin and IL-6, the biomarkers directly measured in our population. This avoids relying on external correction algorithms not supported by our dataset.

By grounding the analysis exclusively on measured data, the interpretation no longer depends on the more extreme values previously generated with BRINDA.

**d. You then state in lines 307-309 that 27.5% of anemia is associated with ID, directly contradicting the paragraph before. Clarify.**

**ANSWER:** Thank you for pointing out this apparent inconsistency. The two values refer to different epidemiological metrics, which we now clarify explicitly in the manuscript:

1. 11% refers to the prevalence of iron deficiency (ID) in the total study population, that is, the proportion of all children with ferritin <12 ng/mL, regardless of anemia status.
2. 27.5% refers to the attributable fraction of anemia due to ID, that is, the proportion of *anemia cases* (not the whole population) that can be attributed to iron deficiency (ID).

These two measures are conceptually distinct:

- Prevalence describes how common ID is in the full sample.
- Attributable fraction describes the proportion of anemia cases specifically caused by ID among children who are anemic.

We have revised the text to clearly differentiate these concepts and avoid confusion, and we now state explicitly:

“*Although iron deficiency was present in 11% of the total population, among children with anemia, 27.5% of cases were attributable to iron deficiency*.”

This clarification ensures that the findings are internally consistent and correctly interpreted.

**Observation 6.** **Lines 270-271, unclear why you included an equation here. If describing correlation, using R^2^.**

**ANSWER:** Thank you for this observation. We agree that including the regression equation was unnecessary and did not add clarity to the description of the associations. As suggested, we removed the equation and now report only the correlation statistics using R² values.

The revised text reads as follows:

“*No significant correlation was observed between serum IL-6 and serum ferritin (R² = 0.0004; p > 0.05), and no correlation was found between serum IL-6 and serum hepcidin levels (R² = 0.072; p > 0.05)*.”

This modification improves readability and aligns the reporting of associations with standard epidemiological practice.

**Observation 7.** **The sentence in lines 292-294 is confusing. As written, it sounds as if these proportions are from an original report from the WHO and then the updated WHO guidelines, but the updated guidelines did not do attribution of causes. If this 26.2% comes from your results, then be clear about that and do not add a citation to this number. Also, if you are specifying all causes besides ID, it should be 26.6%+45.9% based on your results.**

**ANSWER:** Thank you for this important observation. We agree that the previous wording could be interpreted as if the updated 2024 WHO guidelines provided new estimates of the causes of anemia, which is not the case. The updated WHO guideline only revised hemoglobin thresholds and did not redefine etiologic attributions.

To avoid this misunderstanding, we rewrote the paragraph to clearly state that the proportions (50.2% and 26.6%) come from our own analyses, using the old and new WHO Hb cutoffs, respectively. The revised text now reads:

“*Using the old WHO hemoglobin cutoff, 50.2% of anemia cases in our sample were attributed to non–iron deficiency causes. In contrast, when applying the updated WHO hemoglobin threshold, 26.6% of anemia cases were attributed to non–iron deficiency causes in our study population. These values reflect changes in the number of children classified as anemic under different Hb thresholds, not differences in the underlying etiologic distribution of anemia causes*”

This revision:

- Makes it explicit that both values (50.2% and 26.6%) originate from our results, not from WHO reports.
- Removes the citation that could imply external attribution estimates.
- Reflects the change in the denominator (the group classified as anemic) when applying the new WHO 2024 guideline.
- Avoids conflating “non–ID causes” with “all causes other than ID,” which would include inflammation-mediated anemia (45.9% in our study).

**Observation 8.** **In the strengths, you write that you measured inflammatory markers, as plural. But as I understand it you only measured one inflammatory marker, IL-6.**

**ANSWER:** Thank you for noting this. We agree with the reviewer, and this has been corrected.
The revised sentence now reads:

“*Moreover, we measured markers of iron status and a single inflammatory marker (IL-6), which allowed us to estimate the fractions of anemia attributable to iron deficiency and to inflammation*.”

**Observation 9.** **Lines 350-351, the used of the updated WHO guidelines is noted as a strength. This does not seem like a strength to me if the aim of the paper is to compare the old versus new guidelines.**
**ANSWER:** We appreciate the reviewer’s observation. We agree that using the updated WHO 2024 guidelines should not be presented as a methodological strength, given that one of the main objectives of our study was precisely to compare the 2001 and 2024 WHO criteria. Highlighting the new guidelines as a “strength” could therefore be misleading.

In response, this statement has been removed from the manuscript.

The revised strengths paragraph now focuses only on elements that genuinely strengthen the internal validity and relevance of our study, as for example:

- the evaluation of infants across all 13 provinces of the Puno region, covering a broad altitudinal range (600–4,500 m),
- the measurement of iron-status biomarkers and IL-6 concentrations,
- the ability to estimate the attributable fractions of iron deficiency and inflammation using directly measured markers, and
- the study of a highly vulnerable population with a high anemia burden.

This updated framing ensures that the strengths highlighted are truly methodological and contextual, not simply related to the comparison framework itself.

The final text is as follows: *“The strengths of the present study include the enrollment of a population of the southern Peruvian region with a high prevalence of anemia, in which the 13 provinces of the Puno Region have been evaluated covering altitudes of 600 to 4,500 meters…”*

**Observation 10.** **Lines 357-358. I don’t think this is a valid rebuttal to this limitation. You did not use CRP and AGP, limiting the use of the BRINDA method, and the inflammatory marker you did use does not have a validated threshold. This must be included in the limitations.**

**Answer:** We appreciate the reviewer’s clarification. All references to the BRINDA method have now been removed from the manuscript, since its application is not possible without measurements of CRP and AGP.

To address this limitation transparently, we have explicitly acknowledged that the absence of CRP and AGP prevents the use of BRINDA-adjusted inflammation corrections and limits comparability with studies that rely on those biomarkers. The revised text now reads:

“*Furthermore, we cannot perform comparative analyses with studies that apply the BRINDA method (which uses CRP and AGP) because these biomarkers were not measured in our study*.”

In addition, we have also included the reviewer’s point that IL-6 does not have a universally validated threshold for defining inflammation-mediated anemia. This is now clearly stated as a limitation:

“*A further limitation is the use of IL-6, rather than CRP and AGP, to assess inflammation, as no standardized IL-6 cutoff exists for defining inflammation-mediated anemia. This lack of harmonization limits the direct comparability of our results with studies based on CRP/AGP-defined inflammation.”*

**Observation 11.** **Table 4 requires clarification:
a. The row "Anemia associated with ID" (31.3%) is undefined and doesn't correspond to any other reported value. Please clarify what this represents.**

**ANSWER:** Thank you for this comment. The row “Anemia associated with ID” has been removed, as it did not represent an independent category and could create confusion. This value was redundant because all cases of iron-deficiency anemia (IDA) are already included within the total anemia prevalence.

The revised Table 4 now reports only clearly defined and mutually exclusive categories (IDA, inflammation without IDA, inflammation with IDA) and the attributable fractions calculated according to the methods described. This ensures consistency between the definitions in the manuscript and the values displayed in the table.

Table 4. Prevalence of anemia, iron deficiency anemia (IDA), inflammation-mediated anemia and attributable fractions through unadjusted comparisons

|  | 6 to 59  Months  (95% CI) | 6 to 23  Months  (95% CI) | 24 to 59  Months  (95% CI) |
| --- | --- | --- | --- |
| Anemia prevalence | 42.2 (36.7; 47.9) | 46.9 (35.7; 58.3) | 40.6 (34.2; 47.2) |
| IDA prevalence* | 13.2 (9.7, 17.5) | 19.7 (11.7; 30.0) | 10.9 (7.2; 15.6) |
| IDA w/o Inflammation* | 11.0 (7.7; 15.0) | 16.1 (8.8; 25.9) | 9.2 (5.7; 13.7) |
| Inflammation** w/o IDA | 19.4 (15.1; 24.2) | 17.3 (9.8; 27.3) | 20.1 (15.1; 25.9) |
| Inflammation** with IDA | 2.3 (0.9; 4.6) | 3.7 (0.8; 10.4) | 1.8 (0.5; 4.4) |
| Attributable Fraction (AF) | | | |
| AF ID w/o Inflammation | 27.5 (22.5; 32.7) | 34.2 (24.2; 45.8) | 24.7 (19.0; 31.0) |
| AF Inflammation w/o ID | 45.9 (40.2; 51.5) | 36.8 (25.4; 47.2) | 49.3 (42.7; 56.0) |
| Other AF^##^ | 26.6 (21.8; 31.6) | 29.0 (19.6; 39.5) | 26.0 (20.5; 31.8) |

AF for ID was calculated as the prevalence of IDA without inflammation divided by the total anemia prevalence according to the previously described method [25].

#ID was defined when serum ferritin was < 12 ng/mL

*IDA was defined when serum ferritin was < 12 ng/mL and Hb below the WHO cutoff point and

**Inflammation when IL-6 was > 60 pg/mL.

^##^Other AF: unmeasured attributable fraction.

**b. The attributable fractions don't sum to 100%. The text mentions 26.6% "other causes" but this isn't shown in the table.**

**ANSWER:** Thank you for the observation. The attributable fractions have now been recalculated and the category corresponding to “other causes” has been explicitly added to Table 4. As a result, the attributable fractions for each age group now sum to 100%.

We also clarified in the manuscript that this residual category represents unmeasured contributors to anemia, such as genetic conditions, deficiencies of micronutrients not assessed in this study, and environmental exposures, which were not directly evaluated. These factors were therefore included only as a composite “*other attributable fraction or other unmeasured attributable fraction*.”

**c. The opposite age-pattern results between methods (BRINDA: younger children have more inflammation; your IL-6 method: older children have more inflammation) needs explanation.**

**ANSWER:** Thank you for this observation. All analyses based on the BRINDA method have been removed from the manuscript, as the biomarkers required for BRINDA (CRP and AGP) were not measured in our study. Since BRINDA is no longer used in the analysis or discussed in the results, the age-pattern comparison between BRINDA and the IL-6–based approach is no longer relevant to the current version of the manuscript.

**Reviewer #2:** **The majority of recommendation were addressed by the author. A few still lingers, as described below.**

**Observation 12. Table 2 “normal after update cutpoint children” was not corrected in the footnote. Change it to “normal after reclassification”**

**Answer:** Thank you for pointing this out. The footnote for Table 3 has been corrected as recommended, replacing the previous wording with “normal after reclassification,” and the table number has been changed. Table 2 has also been revised accordingly, and all terminology is now consistent with the updated WHO guidance and our reclassification approach.

**Table 2.** Descriptive statistics of the sample of infants and children aged 6-59 months residing in the 13 districts of Puno, Peru.

| **Variables** | | **N (%)** |
| --- | --- | --- |
| Age (months)  6-23  24-59 | | 81 (26.1)  229 (73.9) |
| Age (months)^&^  Sex | | 33.44 ± 14.2 |
|  | Male | 144 (46.5) |
|  | Female | 166 (53.5) |
| Hemoglobin^*^ according to old WHO guideline [3]^&^ | | 10.8 ± 0.1 |
| Hemoglobin^*^ according to new WHO guideline [2]^&^ | | 11.0 ± 0.1 |
| Biomarkers in serum^&^ | |  |
|  | Ferritin (ng/mL) | 21.07 ± 1.5 |
|  | sTfR (mg/L) | 0.75 ± 0.03 |
|  | Erythropoietin (mlU/mL) | 22.3 ± 0.9 |
|  | Hepcidin (ng/mL) | 24.6 ± 1.1 |
|  | Interleukin-6 (pg/mL) | 44.5 ± 1.3 |
| Iron deficiency^#^  Inflammation (50pg/mL)^##^ | | 75 (24.2)  114 (36.8) |
| Inflammation (60 pg/mL)^###^ | | 67 (21.6) |
| Some variables do not add up to 100% due to missing values.  ^&^Quantitative variables (age, hemoglobin and biomarkers) expressed as mean ± SD. * Hemoglobin adjusted according to WHO 2001 or 2024 guidelines. ^#^Iron deficiency: Serum ferritin <12 ng/ml. ^##^Inflammation: IL-6>50 pg/mL. ^###^Inflammation: IL-6>60 pg/mL Anemia was defined when Hb <10.5 g/dl in infants aged 6-23 months and Hb<11 g/dL for children aged 24-59 months. Hemoglobin was adjusted for altitude according to old guidelines [3] or updated WHO guidelines [2] | | |

**Table 3.** Hemoglobin, iron, and inflammatory status in children aged 6-59 months using 2024 WHO anemia guidelines.

| **Biomarkers** | **Normal Hb, (n=155)** | **Anemic, (n=131)** | **Normal Hb**  **after reclassification, (n=24)** |
| --- | --- | --- | --- |
| Hb (g/dL)^¥^ | 12.2±0.1 | 9.7±0.1^*^ | 10.9±0.1^**,#^ |
| Hepcidin (ng/mL) | 23.9±1.7 | 25.3±1.51 | 25.1±1.9 |
| sTfR (mg/L) | 0.7±0.04 | 0.8±0.04 | 0.9±0.1 |
| Ferritin (ng/mL) | 23.5±2.7 | 18.6±1.05 | 19.6±2.9 |
| IL-6 (pg/mL) | 43.2±1.7 | 47.3±2.17 | 37.5±4.5# |
| EPO (mlU/mL) | 21.7±1.5 | 22.3±5.60 | 26.5±2.9 |

Data are reported as mean ± SD. Hb: Hemoglobin; EPO: Erythropoietin; sTfR: Soluble transferrin receptor; IL-6: Interleukin-6. ^¥^Adjusted Hb (updated version) for altitude was calculated using the guidelines from WHO in 2024 [3]. *p<0.001 between normal and anemic children. **p<0.001 between normal and normal after reclassification children. #p<0.001 between anemic and normal after reclassification children.

**Observation 13.** **Figure 1 Although I do understand that it is a matter of choice from the authors, in my perception, severe/moderate/mild reclassification changes are better visualized in a piled bars chart. It will not include the changes in total prevalence though. Also, the percentage values (59%, 46,4%…) should be plotted (this recommendation applies for other charts too).**

**Answer:**

Figure 1. Prevalences of anemia in infants aged 6 to 59 months residing in Puno, by category. Black bar, mild anemia. Black lines bar, moderate anemia. And, gray bar, severe anemia calculated based on old and new WHO guidelines.

Thank you for the suggestion. Figure 1 has been modified according to the reviewer’s recommendation by adopting a stacked bar chart format, which improves visualization of the reclassification into mild, moderate, and severe anemia. Percentage values have also been added to enhance interpretability.

Figure 2 has been retained, as its current layout allows clearer statistical comparisons relevant to our analyses.

Figure 3 has been removed because its information is now fully represented in Table 4, making it redundant.

**Observation 14.** **Figure 2 The legend “Anemia 2001/Anemia 2024” gives the impression that there were a temporal analysis, in different years, when it was just the year of the guidelines. Correct it for clarity.**

**Answer:** Thank you for noting this. The legend has been revised to avoid any suggestion of a temporal comparison. The labels “Anemia 2001 / Anemia 2024” have been replaced with “Anemia according to WHO 2001 guideline” and “Anemia according to WHO 2024 guideline,” making it clear that the distinction refers to the guideline versions rather than different years of data collection.
